# QuickStats

**Published:** 2013-03-01

**Authors:** Jiaquan Xu

**Figure:**
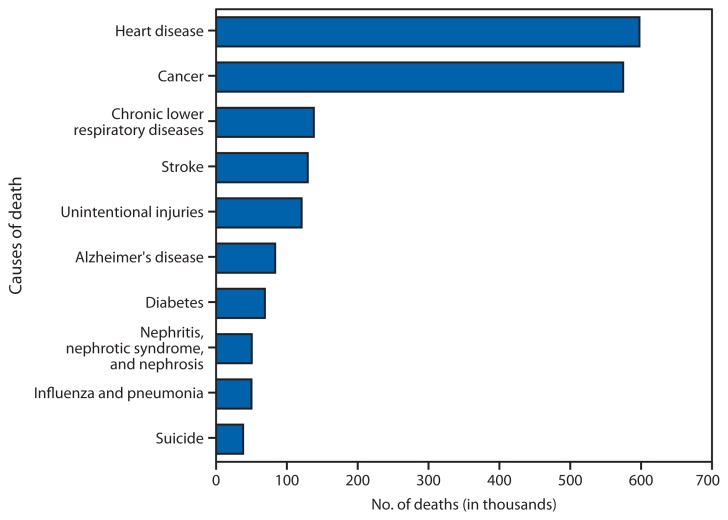
Number of Deaths from 10 Leading Causes — National Vital Statistics System, United States, 2010

In 2010, a total of 2,468,435 deaths occurred in the United States. The first two leading causes of death, heart disease (597,689 deaths) and cancer (574,743), accounted for nearly 50% of all deaths. In contrast, the other leading causes accounted for much smaller percentages, ranging from 5.6% (138,080 deaths) for the third leading cause of death, chronic lower respiratory disease, to 1.6% (38,364) for suicide, the 10th leading cause of death. All other causes combined accounted for 25% of the deaths.

**Source:** Murphy SL, Xu JQ, Kochanek KD. Deaths: final data for 2010. Available at http://www.cdc.gov/nchs/data/dvs/deaths_2010_release.pdf.

